# Editorial: Molecular mechanism in the development and pathogenesis of fungi

**DOI:** 10.3389/ffunb.2023.1231925

**Published:** 2023-06-13

**Authors:** Jeyaprakash Rajendhran, Yingzi Yun, Wei Tang, Ya Li

**Affiliations:** ^1^ Department of Genetics, School of Biological Sciences, Madurai Kamaraj University, Madurai, India; ^2^ Fujian Agriculture and Forestry University, Fuzhou, China

**Keywords:** fungal pathogen, molecular mechanisms, host-pathogen interaction, phytopathogen, proteomics

Fungi are a diverse group of eukaryotic organisms that play crucial roles in various ecosystems. Fungi are exceptionally diverse, with over 144,000 known species and potentially millions more yet to be discovered (Hawksworth and Lücking, 2017). They are found in almost all habitats on Earth (Frąc et al., 2018). While many fungi are beneficial, some are pathogenic to plants, animals, and humans. Fungal pathogens can invade and colonize host tissues, leading to various infections with varying degrees of severity. The pathogenesis of fungi involves several key steps. Fungal pathogens typically enter the body through inhalation, ingestion, or direct contact with the skin. Adherence to host tissues is the initial step in establishing an infection. Fungal cells have specific surface molecules or adhesins interacting with host cell receptors or extracellular matrix components, promoting fungal attachment. Fungal pathogens also form biofilms, which protect them from host immune defenses and antimicrobial agents. Fungal biofilms are commonly associated with chronic or recurrent infections and are more resistant to treatment.

The host’s immune system is critical in recognizing and eliminating fungal pathogens. However, fungi have developed several strategies to evade or suppress the host’s immune response. Certain fungal pathogens produce mycotoxins, which can damage host cells and disrupt cellular processes, leading to tissue damage, inflammation, and immunosuppression. Fungal pathogens may produce immunomodulatory molecules that interfere with the host’s immune response. Fungi also exhibit morphological transitions in their life cycle. These transitions often involve changes in structure and reproductive strategies, such as spore formation, encystment, filamentous growth, and mycelial aggregation. These transitions enable fungi to adapt to changing environmental conditions, including nutrient availability and host interactions. Understanding the molecular mechanisms of fungal pathogenesis is crucial to develop against fungal infections. Targeting specific host-fungal interactions may help to develop novel antifungal therapeutic approaches.

In this Research Topic, three research articles are focused on the molecular mechanisms of fungal phytopathogens. One is focused on fungal pathogenicity in amoebae trophozoites and murine macrophages. Liu et al. have reported the role of NADPH oxidases (NOXs) in the pathogenicity of the phytopathogen *Colletotrichum gloeosporioidesn* on *Hevea* leaves. The NOXs are required for the polarized growth of hyphal tips and the pathogenicity of phytopathogens to the host plant. Previously, this research group has identified two NOX components, CgNOXA and CgNOXB, and a regulatory protein CgNOXR in *C. gloeosporioides*. In this study, they have shown that CgNOXB and CgNOXR are essential for the polarization of actin organization in the hyphal tip, cell wall component deposition, and appressorium formation. Further, authors have demonstrated that NOXs remodel the actin cytoskeleton during pathogenicity.

Tsuji et al. have shown the regulation of pathogenicity through RNA silencing in the plant pathogen, *Bipolaris maydis*, that causes corn leaf blight disease. GTP-binding proteins, known as septins, play essential roles in morphological development and pathogenicity in fungi. In this study, authors have demonstrated the existence of “ascus dominance” in *B. maydis* mediated by meiotic silencing by unpaired DNA (MSUD). Ascus dominance was identified by crossing septin mutants and the wild-type strain of *B. maydis*. Further, an RNA-dependent RNA polymerase (RDR1) involved in MSUD was identified. By comparing the genetic crosses using the wild-type and the *Δrdr1* strain with septin mutants, authors have proposed that ascus dominance is caused by RNA silencing triggered by an unpaired gene, and septin genes were affected by this silencing. Further, the Δ*rdr1* strain can be used to study the localization of septins using fluorescent proteins in the ascosporogenesis of *B. maydis*. Hitherto, MSUD has been reported only in *Fusarium graminearum* and *Neurospora crassa*, which belong to Sordariomycetes. This study showed that MSUD is also functional in *B. maydis* belonging to Dothideomycete for the first time.

Using a proteomics approach, Chen et al. have reported the molecular mechanism of MoPer1-mediated regulation in *Magnaporthe oryzae*, causing rice blast disease. In fungi, many cell wall proteins must be anchored by glycosylphosphatidylinositol (GPI) before binding to the cell wall. Previously, authors have shown that MoPer1, a GPI anchoring essential factor, has critical roles in the growth, pathogenicity, and conidiogenesis of *M. oryzae.* This study compared the glycoproteins from the *ΔMoper1* mutant and the wild-type strain Guy11 by quantitative proteomic analysis employing mass spectrometry. They found 431 proteins were differentially expressed with significant changes in levels. Of these, 14 proteins are associated with the growth, development, and pathogenicity of *M. oryzae*.

Ferreira et al. reported the conserved role of fungal cell wall mannosylated components in the adhesion, invasion, and survival within the amoeba trophozoites and murine macrophages. Authors have compared the high-affinity ligands for mannosylated fungal cell wall components expressed on the surface of amoebas and murine macrophages. Mannose-purified surface proteins (MPPs) from both phagocytes showed binding to isolated mannose/mannans and mannosylated fungal cell wall components. Macrophage MPPs showed more affinity and intense binding when compared to the amoeba receptors. Computational analyses showed highly conserved regions between amoeboid and murine receptors, suggesting a possible convergent evolution of pathogen recognition mechanisms in amoeba and murine macrophages.

## Author contributions

All authors listed have made a substantial, direct, and intellectual contribution to the work and approved it for publication.

## References

[B1] Frąc M. Hannula S. E. Bełka M. Jędryczka M. (2018). Fungal biodiversity and their role in soil health. Front. Microbiol. 9. doi: 10.3389/fmicb.2018.00707 PMC5932366 29755421

[B2] Hawksworth D. L. Lücking R. (2017). Fungal diversity revisited: 2.2 to 3.8 million species. Microbiol. Spectr. 5 (4). doi: 10.1128/microbiolspec.FUNK-0052-2016 PMC11687528 28752818

